# An algorithm for drug discovery based on deep learning with an example of developing a drug for the treatment of lung cancer

**DOI:** 10.3389/fbinf.2023.1225149

**Published:** 2023-11-09

**Authors:** Dmitrii K. Chebanov, Vsevolod A. Misyurin, Irina Zh. Shubina

**Affiliations:** ^1^ Department of Molecular Biology of Cancer, BioAlg Corp., Covina, CA, United States; ^2^ The Russian Melanoma Professional Association (Melanoma.PRO), Moscow, Russia

**Keywords:** drug discovery, artificial intelligence, deep learning, modeling, simulation, chemoinformatic

## Abstract

In this study, we present an algorithmic framework integrated within the created software platform tailored for the discovery of novel small-molecule anti-tumor agents. Our approach was exemplified in the context of combatting lung cancer. In the initial phase, target identification for therapeutic intervention was accomplished. Leveraging deep learning, we scrutinized gene expression profiles, focusing on those associated with adverse clinical outcomes in lung cancer patients. Augmenting this, generative adversarial neural (GAN) networks were employed to amass additional patient data. This effort yielded a subset of genes definitively linked to unfavorable prognoses. We further employed deep learning to delineate genes capable of discriminating between normal and tumor tissues based on expression patterns. The remaining genes were earmarked as potential targets for precision lung cancer therapy. Subsequently, a dedicated module was formulated to predict the interactions between inhibitors and proteins. To achieve this, protein amino acid sequences and chemical compound formulations engaged in protein interactions were encoded into vectorized representations. Additionally, a deep learning-based component was developed to forecast IC_50_ values through experimentation on cell lines. Virtual pre-clinical trials employing these inhibitors facilitated the selection of pertinent cell lines for subsequent laboratory assays. In summary, our study culminated in the derivation of several small-molecule formulas projected to bind selectively to specific proteins. This algorithmic platform holds promise in accelerating the identification and design of anti-tumor compounds, a critical pursuit in advancing targeted cancer therapies.

## Introduction

The persistent challenge of effectively treating cancer patients remains a matter of utmost significance as issues related to relapses and drug resistance in antitumor therapies continue to pose unresolved hurdles. Addressing these challenges necessitates the development of novel therapeutic agents that exhibit superior efficacy compared to those already sanctioned. However, this pursuit of innovation inevitably contributes to escalated research and production costs.

To surmount these obstacles, the realm of bioinformatics has embraced the power of computational methodologies, offering a promising avenue to revolutionize drug discovery. Notably, deep learning technologies have garnered substantial success across diverse scientific and industrial domains, enabling the resolution of intricate problems through an unparalleled degree of abstraction unattainable by the human mind.

In this context, the integration of machine learning models emerges as a transformative solution for identifying potential candidates for novel drugs. A prime example lies in harnessing machine learning to predict the therapeutic attributes of molecular compounds, thereby facilitating systematic exploration within vast chemical libraries. Furthermore, the predictive capabilities of machine learning extend to deciphering intricate drug–protein interactions, thereby unveiling precise protein targets and potential inhibitor molecules.

An additional facet of machine learning’s prowess lies in its capacity to forecast the outcomes of pivotal IC_50_ experiments. By assimilating genomic expression profiles of cellular lines and molecular structures, these models can prognosticate the feasibility of achieving established IC_50_ values. This emulation of *in silico* cellular experiments showcases the potential to streamline research efforts and augment the drug discovery process.

**FIGURE 1:**
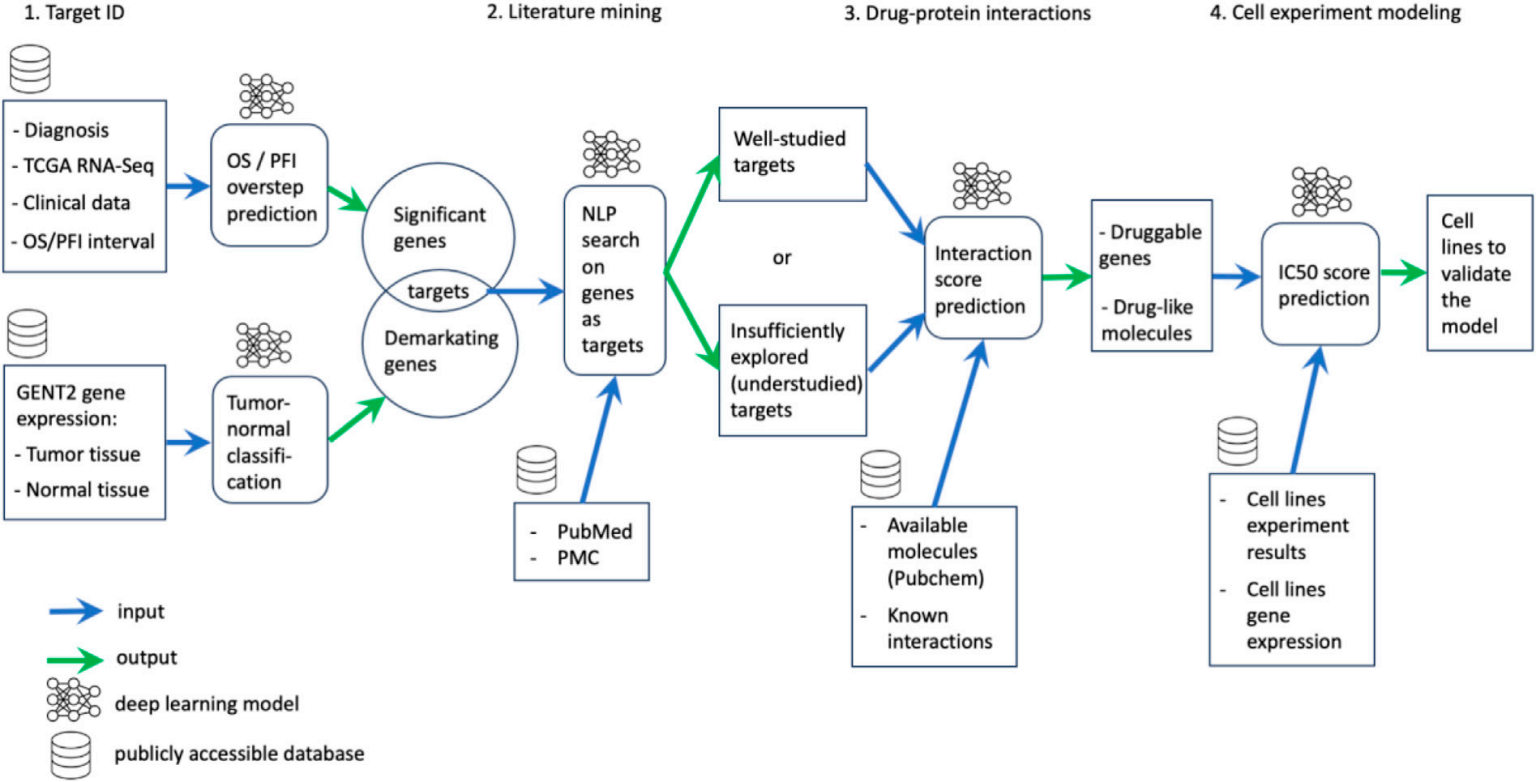
Overview of the overall pipeline structure.

This article delves into the application of machine learning methodologies throughout the drug discovery process, encompassing stages such as target identification, literature retrieval, and selection of molecular inhibitors guided by target interactions, as well as the strategic planning and predictive modeling of preclinical studies (Figure 1).

### Target identification

The foundational step in drug discovery hinges upon the precise delineation of therapeutic targets. The multifaceted nature of this endeavor necessitates adherence to several imperative criteria. Primarily, targets should exhibit a degree of specificity that highlights disparities between tumor and normal tissues. Furthermore, their involvement in tumor cell survival pathways is paramount. Equally significant is their amenability to small molecule-based interventions. Notably, our research advances the proposition that altered genes, underpinning increased disease aggressiveness and decreased overall survival, hold promise as prime targets. This aligns with the overarching objective of machine learning-driven target identification—an effective prediction of overall survival and relapse-free interval through the discerning selection of indispensable genes while simultaneously accounting for distinctive expression profiles in comparison to normal tissues.

### Optimizing target gene selection

A nuanced challenge arises from genes implicated in pivotal cellular processes that are shared between tumor and normal cells. The intricate balance of targeting such genes mandates a judicious approach. To this end, we have incorporated a refined strategy. Leveraging comprehensive gene expression data from tumor and normal tissues, we discriminate against genes displaying marginal expression differences. Employing deep learning algorithms, we delineate genes that decisively demarcate tumor and normal tissues, thus augmenting the precision of target gene selection.

### Harnessing deep learning for literature mining

Beyond the confines of experimental data, deep learning extends its scope to the vast expanse of the published literature. Our research capitalizes on this potential, streamlining decision-making processes by assimilating insights from a meticulously curated repository of scientific articles. A dedicated tabulated summary of findings from a PubMed and PMC search fortifies the arsenal of tools for selecting promising molecular inhibitors.

### Predictive modeling for drug–protein interactions

A pivotal axis of drug discovery revolves around predicting the interaction dynamics between drug molecules and target proteins. We introduce an innovative deep learning model, integrating intricate protein and drug molecule information. This model prognosticates the impact of drug molecules on target proteins, ushering in a refined selection process. This stage inherently winnows the gamut of potential targets, eliminating candidates whose inhibition feasibility or binding efficacy raises concerns.

### Navigating toward preclinical trials

Transitioning toward preclinical studies demands the emulation of cellular experiments, a crucial precursor to laboratory validation. Deliberations encompassing cell lines that accurately mirror real-world conditions are pivotal. Our investigation extends to prognosticating the likelihood of compounds from prior stages attaining IC_50_ concentrations within select cell lines, paving a trajectory toward informed preclinical trial design.

In the synthesis of these insights, our study embarks on a journey through the intricate tapestry of machine learning-driven drug discovery in oncology. From the meticulous identification of target genes to the finesse of molecular interaction prediction and the refinement of preclinical trial design, the amalgamation of cutting-edge techniques and comprehensive data analyses presents a formidable paradigm shift in the pursuit of effective therapeutics.

## Materials and methods

### Target identification

To assess the effect of gene expression on disease prognosis, we used the data on gene expression from the open database TCGA https://gdc.cancer.gov (Cancer Genome Atlas Research Network et al., 2013). In this section, gene expression data (RNA-Seq) were acquired and subsequently subjected to a normalization procedure. Normalization was performed by aligning the expression values to the reference levels represented by the control gene GAPDH, commonly referred to as a “housekeeping” gene. This process was undertaken to facilitate the integration of newly acquired data into the database. The data on overall survival (OS), progression-free interval (PFI), and the same parameters within the follow-up period were derived from this database. The problem of OS prediction was successfully solved in our previous study (Chebanov et al., 2021).

The essence of applying machine learning in this context can be summarized as follows: a dataset is prepared comprising features that include cancer-associated genes and patient medical history data. The target variable is a binary outcome representing whether the patient surpasses the median PFI or OS value for the whole dataset. A model is constructed using a multi-layer perceptron within the Python environment utilizing the Keras library. Upon achieving satisfactory training quality, the most influential features affecting the prediction are extracted from the dataset.

As an example, we selected the diagnosis of lung cancer and extracted a cohort of 514 patients with this diagnosis from the database.

In order to minimize training noise, we specifically curated a dataset containing genes that are integral to tumor-associated signaling pathways, as defined by the KEGG resource. This meticulous curation resulted in the inclusion of a total of 1,821 genes (Kanehisa and Goto, 2000).

To begin, we initially trained the algorithm utilizing a dataset comprising 514 patient records. However, following a rigorous five-fold cross-validation procedure, we observed that the mean ROC-AUC values were 0.69 (0.61–0.74) for overall survival (OS) and 0.61 (0.54–0.69) for progression-free interval (PFI). These results collectively signify a level of predictive performance that falls short of expectations, thus indicating the need for further refinement and enhancement of our predictive model. The reason is that it was a very small dataset for the application of neural networks, even taking into account more than 1,800 features.

To avoid this, we generated additional data comprising 50,000 synthetic patients by applying a generative adversarial network (GAN) to the tabular data on the existing 514 patients. GAN technology has been successfully used in various industries, such as image generation (Goodfellow et al., 2014). To use this methodology, we leveraged the Python SDV library (Patki et al., 2016) with a specific focus on utilizing the CTGAN module (Xu et al., 2019).

### Optimizing target gene selection

To refine the list of potential targets, we trained a deep learning algorithm to classify tissue into healthy and tumor categories based on gene expression. Subsequently, we ranked the features of the original dataset by importance, and genes with the greatest influence on the prediction were identified as more probable candidates for targeting as they contribute more significantly to distinguishing tumors from healthy tissue.

Gene expressions for tumor-normal data were taken from the GENT2 database (Park et al., 2019). This database comprises information on 68,287 samples of patients’ tissues and cell lines for all the diagnoses, of which 58,041 were tumor samples and 10,246 were normal samples.

#### Literature mining

We developed a deep learning-based tool for named entity recognition (NER) based on the technology of natural language processing (NLP), with the help of the Python library Biopython, for which we trained the NLP algorithm on the abstracts of articles labeled by hand. We identified the name of the gene or protein of interest and the name of its inhibitor. We used the BERT algorithm (Devlin et al., 2019) as the basis. We applied the fine-tuning procedure for this algorithm, which included training on the dataset of the labeled abstracts with the BRAF gene and its inhibitors.

The created algorithm helped achieve 98% accuracy of prediction.

#### Drug–protein interactions

We obtained the drug data from the open DrugBank database (Wishart et al., 2017). This database contains data about drugs in combination with targets, including the protein that the drug is targeting, as well as structural representations of the molecules. We have selected only those small molecules for which there is a representation in the SMILES format. We needed two types of data to prepare the dataset: a target protein and a structural representation of the molecule.

As a result, the data array included positive examples with 19,256 interactions for 5,769 drugs and comprised 4,104 unique proteins encoded by 3,516 genes.

A challenge was to find negative examples for the training set. Researchers solve the problem in different ways: for instance, Wang et al. (2018) reported that they randomly selected negative drug–target pairs with no interaction data. Researchers of another study also obtained negative examples by extracting pairs with no interaction data while randomly choosing the number of examples equal to the number of positive examples (Wang et al., 2020). Some authors predicted the absence of an interaction by the algorithm (Liu et al., 2015).

We analyzed the STITCH database (Szklarczyk et al., 2016), which contains scores of interactions between proteins and compounds.

To determine which score to regard as negative, we correlated interactions from the STITCH database with the DrugBank database, which included only pairs with a positive score. Thus, we expected we could understand which value to consider a “positive speed.”

**FIGURE 2:**
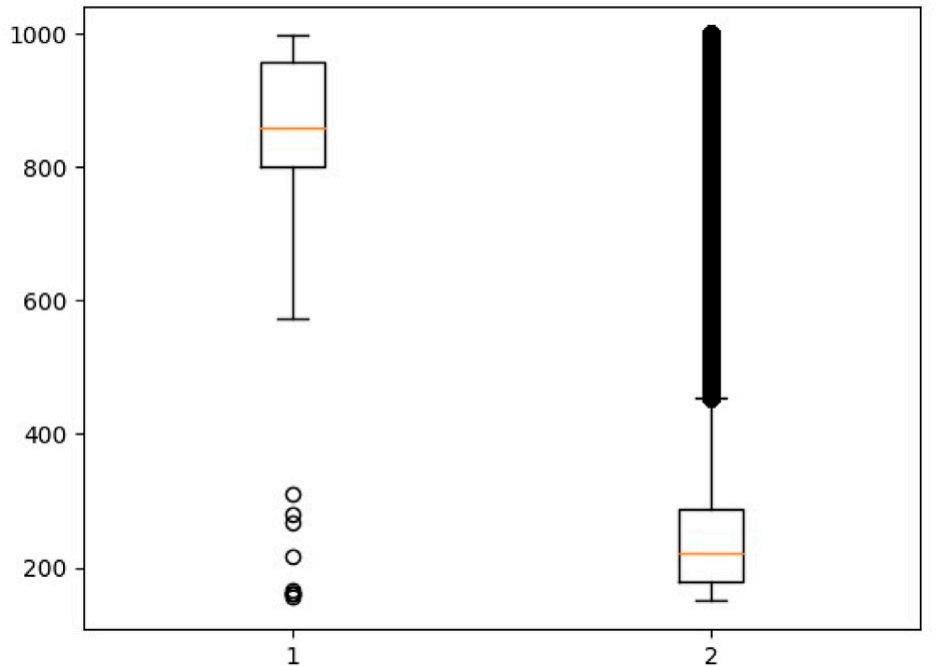
1–Strength of the interaction between the compound and the protein, described in both databases (STITCH and DrugBank). 2–Boxplot for all values from the STITCH database.

The left part of Figure 2 presents a boxplot for pairs that are present in both databases (STITCH and DrugBank); therefore, they are considered positive. The right part presents a boxplot for all values from the STITCH database. Thus, it is evident that the range of positive rates does not intersect with the main range of data from the database of all interactions and is an outlier for it.

We used the lower quartile of interaction rates to form a sample of non-protein-binding drugs. We sorted the compounds of the obtained data, according to the number of known interactions with proteins, and selected the 50,000 most common compounds.

Amino acid sequences were obtained from the UniProt database (Martin et al., 2021). We presented them in vector form using the approach described elsewhere (Asgari and Mofrad, 2015), where the authors vectorized all possible amino acid triplets (8,000) in the form of a 100-dimensional vector, and thus, the vector representation of any protein consisting of these triplets was equal to their vector sum.

We also presented the compounds included in the dataset in a vector form and in the form of 100-dimensional vectors, using the embedding approach of natural language processing technologies and implemented in the RDKit (RDKit), mol2vec (Jaeger et al., 2018), and word2vec (Mikolov et al., 2013) libraries for Python3 language.

To search for candidate molecules, we experimented with predicting interactions for pairs of genes and compounds of all possible ones.

We used all the molecules from the PubChem library, which had representations in the form of SMILES (23 million in total). They were presented in a vector form similar to that in the learning process. Amino acid sequences of the encoding proteins were obtained from the UniProt database for 12 genes that we received earlier. The prediction result was the DPI probability.

### Preclinical trial modeling

The dataset for cell experiment emulation was formed using the data on gene expression profiles of the cell lines from the Cancer Cell Line Encyclopedia (CCLE) database and compound sensitivity data for cell lines from the Genomics of Drug Sensitivity in Cancer (GDSC) database (Barretina et al., 2012; Yang et al., 2013). We selected only lung cancer cell lines. A total of 11,330 interactions were obtained for 122 drugs and 32,000 genes in 106 cell lines.

Then, a study was performed for the prediction of these 106 lines’ interactions with potential inhibitors. We substituted each of the 2,921 candidate inhibitors in turn and predicted the success of the IC_50_ test. After that, we selected all molecules with a probability of above 0.9, achieving IC_50_ from the results in the A549 and CALU1 cell lines. These cell lines were chosen due to the most frequent use of these lines in various studies of lung cancer.

The study resulted in the obtained data on the structure of 37 molecules with potential toxicity for lung cancer cells. We performed visualization in our own module and found that the resulting molecules were large and consisted of many repeating structural elements of radicals. Therefore, we decided to isolate the active parts of the molecule for further analysis. As a result, another 15 molecules, after their decomposition, were added to the initial set of 37 molecules.

In the next step, we planned to test the selectivity of the obtained molecules in all 1,018 cell lines. We designed a similar experiment to predict the IC_50_ value.

## Results

### Target identification

We generated 50k patients’ data to reach the ROC-AUC value equal to 0.73. We used the Lasso linear regression algorithm with a 5-fold cross-validation to determine the significance of the features. The result of each experiment was obtained as a list of genes ordered by decreasing impact on the effect. We combined the lists of genes obtained in the experiments with OS and with PFI.

Consequently, we identified 36 genes whose expression correlates with compromised survival indicators in individuals diagnosed with lung cancer. Table 1 presents some characteristics of these genes.

**TABLE 1:** Biological features of the discovered genes.

Gene	Type of encoded protein	Relationship with various processes in the cells or the organism
GNB3*	Signal	Obesity
CHRM1*	Receptor and signal	Regulation of nerve impulses
SHC4***	Signal	Proliferation and apoptosis
FKBP4****	Signal	Immunoregulation
IL17B*	Cytokine	Immunoregulation
ATP6V1E2*	Membrane transporter	ATP synthesis
*FASLG*	Receptor and signaling	Proapoptosis
DKK4****	Signaling	Proliferation, stemness, and chemoresistance
GDF6****	Signaling	Growth factor
*GP6*	Structural	Platelet aggregation
WNT6**** and WNT8B****	Signaling	Differentiation
*HMOX1*	Enzyme	Respiration
LEF1****	Transcription factor	Differentiation and morphology
*ATP1A4*	Membrane transporter	ATP synthesis
ACVR2A***	Receptor and signal	Growth activator
SMAD9***	Signal	Proliferation
*CUL1*	Complex	Protein utilization and cell cycle control
*KRT10*	Structural	Cytoskeleton
*PIAS4*	Regulator	Blocks STAT4
FSHR***	Receptor	Proliferation
CCNA2**	Regulator	Cyclin
RPS6KA4***	Transcriptional CSF2	Proliferation
factor***	Cytokine	Proliferation
EFNA3***	Signaling	Proliferation
*KRT24*, *KRT27*, *MYL10*, and *MYLPF*	Structural	Cytoskeleton
ITGA3*	Structural	Adhesion
ZFYVE9***	Transcription factor	Proliferation
ATP6V1G3*	Complex	Protein
*HEY1*	Transcription factor	Differentiation
PIK3R6****	Receptor and signal	Unknown
BIRC3***	Complex	Inhibitor
GHSR*	Receptor and signaling	Obesity

At the stage of implementation of the tumor–normal filter, deep learning was performed according to the aforementioned method with the ROC-AUC indicator of 0.83. A total of 4,912 genes were selected in the process of determining the significant features. The genes with the expression associated with distinguishing a tumor tissue from the normal tissue were isolated from the previously found 36 genes with the help of the obtained list of genes. These 12 genes were *DKK4*, *GP6*, *LEF1*, *CUL1*, *KRT10*, *PIAS4*, *FSHR*, *MYLPF*, *EFNA3*, *ZFYVE9*, *GHSR*, and *MYL10*.

### Literature mining

As a result of automated literature mining inhibitors, we obtained Table 2, which presents the number of articles published in response to various requests for each of the 12 genes of interest. Such a table will help draw a conclusion about the studying extent of the gene as a target.

**TABLE 2:** Results of the NLP search for keywords associated with the studied genes and their inhibitors. Tags: green—low-molecular weight inhibitor that triggers apoptosis , blue—protein , and red—toxin .

Gene	Gene + ‘target'	Gene + ‘cancer'	Gene + ‘lung cancer'	Gene + ‘phase'	Gene + ‘drug'	Gene + ‘approval'	Gene + ‘FDA'	Total mentions	Inhibitors
*LEF1*	9,661	9,138	4,301	4,962	4,771	2,129	665	35,627	Imatinib , wnt10b , dlx3 , sb203580 , ex527 , dasatinib , t0070907 , and sb431542
*CUL1*	4,854	4,245	1,823	3,629	2,518	634	374	18,077	fbx4 , selumetinib , fbxo7 , fbxo31 , fbxo21 , fbxo4 , and fbxw7
*FSHR*	2,143	2,150	466	1,657	1,752	752	205	9,125	Sunitinib , uk5099 , and clxbpa
*GP6*	1,385	1,977	460	1,023	770	766	199	6,580	ono1714
*GHSR*	1,308	926	365	828	1,224	382	417	5,450	Gefitinib , pd98059 , pd90859 , and sb203580
*KRT10*	934	998	388	700	604	320	75	4,019	r115866 and sb431542
*PIAS4*	810	792	319	505	427	127	53	3,033	nur77 , pax8 , foxm1b , trim32 , and zif268
*DKK4*	652	661	339	312	368	139	48	2,519	
*EFNA3*	542	541	328	272	318	141	28	2,170	
*MYLPF*	301	251	98	192	168	90	17	1,117	
*ZFYVE9*	166	155	82	85	75	46	12	621	
*MYL10*	73	74	33	50	28	35	2	295	

However, some references may not mean that there is a direct connection between the name of the gene and the drug used. In other words, they may not be related to it in terms of inhibition but are simply mentioned in a similar context.

As a result of the NLP search, we added the right column to the table, which lists all inhibitors of a certain gene. These data allow a researcher to make a decision on the basis of the available number of inhibitors for each of the genes under consideration.

### Drug–protein interactions

As a result, a dataset was obtained from 118,379 pairs, including 19,250 pairs describing the compounds bound to proteins and 99,129 precedents describing non-protein-bound compounds.

**FIGURE 3:**
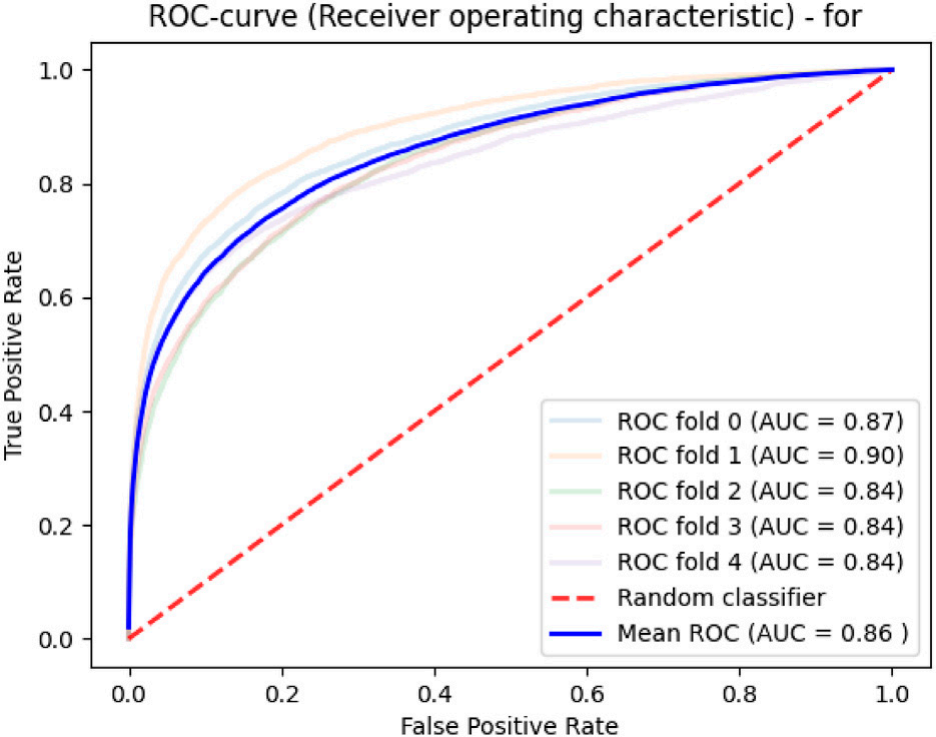
Model quality for predicting drug–protein interactions.

Deep learning was applied in a similar way, as in the previous approach. ROC analysis of learning quality allowed us to obtain an average area under the curve of 0.86 (Figure 3).

After search for candidate molecules, we received 160,000 pairs with an interaction probability over 0.99 and 2,921 pairs with an algorithm predicted probability of 1.0.

The following distribution by the inhibited genes was obtained for these 2,921 potential inhibitors (Table 3).

**TABLE 3:** Results of predicting the inhibitors for the target genes.

Gene	Number of inhibitors found
*LEF1*	639
*MYL10*	524
*FSHR*	461
*EFNA3*	385
*GHSR*	279
*CUL1*	248
*GP6*	190
*DKK4*	134
*PIAS4*	61

### Preclinical trial modeling

**FIGURE 4:**
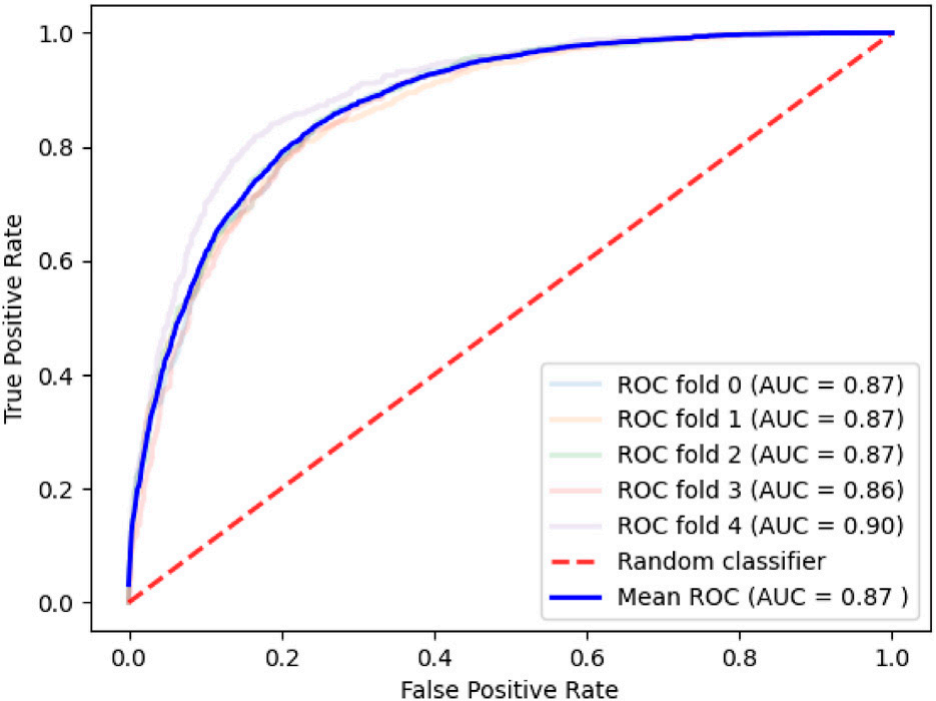
Quality of the model designed to assess the likelihood of reaching the compound IC_50_ data on lung cancer cell lines.

During the cell experiment emulation, the algorithm for determining the importance of the features selected 129 genes. The characteristics of the proteins encoded by the revealed genes are presented in Table 4. ROC is shown on Figure 4.

**TABLE 4:** Characteristics of proteins encoded by the revealed genes.

Encoded proteins	Genes
Structural	*LAMB4*, *PDLIM2*, *C1QC*, *SPATA48*, *CDC42SE1*, *UPK3B*, and *APOBEC3*
Inhibitor	*APOBEC3*, *HTN1*, *APOC1*, *CST5*, and *MRGPRX2*
Metabolism	*MGLL*, *UPB1*, *PPIF*, *AMPD1*, *ESYT2*, *RAB30*, *SLC40A1*, *LHFPL2*, *GALNT14*, *TENT5B*, *PADI4*, *FABP6*, *AKR1B10*, *LIPK*, *AWAT1*, *GAPDHP45*, and *CCDC71L*
Energy	*SCN4A*, *SMOX*, *SLC34A1*, *ATP10A*, and *SLC12A8* membrane
Receptor	*TSPAN9*, *GPRC5A*, *OXT*, *ANXA10*, *ARTN*, *IL37*, *GNG11*, *EPB41L4A*, *OR11HGU1*, *GYAMC*, *UFCAM3*, *FKBP2*, *CCR4*, *OR10J5*, *OR1D2*, *TNFAIP2*, *ANGPTL5*, *TMEM207*, *TRBV6-5*, *TRAV16*, *OMP*, and *FBXW7-AS1*
Kinase	*PRKY*, *SERTAD2*, and *RN7SKP257*
SH3BP1	*SH3BP1* and *BCL2L1*
Transcription factor	*HOXA3*, *AGFG2*, *NKX3-1*, *MBD3L1*, *GBP6*, *AHNAK2*, and *ZNF680P1*
Pseudogene	*TPRXL*, *OR5AZ1P*, *TTTY2*, *GNL3LP1*, *HIGD1AP16*, *RNU2-37P*, *RN7SKP172*, *KRT18P27*, *C1DP3*, *USP21P1*, *ABCD1P4*, *LINC01529*, *LINC01209*, *LINC01433*, *FDPSP7*, *RPL4P2*, *DPYD-AS1*, *MTND6P24*, *LINC00892*, *RPS24P6*, *LINC01731*, *LINC01440*, *LINC00601*, *LINC00993*, *HS6ST2-AS1*, *MCCD1P1*, *YRDCP2*, *HIGD1AP15*, *NRBF2P3*, *RPS13P4*, *RN7SL589P*, *RN7SL573P*, *VPS26AP1*, *RN7SL454P*, *LINC02150*, *LINC02014*, *SALL4P1*, *AACSP1*, *IGHV3-52*, *LINC02700*, *MRGPRF-AS1*, *LBX2-AS1*, *LINC01580*, *LINC00524*, *NDUFB4P11*, *CYCSP2*, *TBC1D3P5*, *RDM1P2*, *RDM1P1*, *ACTBP9*, *NTF6A*, *OR7E16P*, *VN1R80P*, *IMMP1LP3*, and *RDM1P4*

At the cell experiment emulation stage, we chose interactions with a probability of at least 0.9 from the data on the forecast. Molecules were selected that acted on the minimum number of lines with a probability higher than a given one, i.e., those with the highest specificity.

As a result, five small molecules were selected. The certain cell lines used for validation are “A549,” “NCIH23,” “NCIH460,” “NCIH1299,” “HCT116,” “AMO1,” “PC3,” and “CAPAN1.”

## Discussion

During the initial phase of target discovery, approximately 36 genes were meticulously chosen for further investigation. Interestingly, a few genes appeared to be associated with WNT signaling (*DKK4, LEF1, WNT6*, and *WNT8B*), with BMP (*SMAD9*), and TGF (*ACVR2A, GDF6*, and *ZFYVE9*). The study has found various components of the cytoskeleton and membrane proteins responsible for the transfer of various molecules. Potentially, each of these genes encodes proteins suitable for targeted lung cancer therapy.

Four of the genes found at the stage of tumor–normal filter encode cytoskeletal proteins. Notably, the processes of tumor invasion and metastasis are often registered by the time of diagnosis in patients with lung cancer (Kuo et al., 2021). These features of the tumor are mediated by the developed cytoskeleton in the tumor cells. Thus, it is not surprising that there is a correlation between the expression of cytoskeletal proteins and a decrease in the overall survival of patients with lung cancer.

One of the key considerations in deciding to use artificial intelligence algorithms for drug discovery is the reliability of the results obtained through their utilization. Similar to mathematical modeling across various industries, there is a significant possibility of forecast results not being validated in reality. This arises from the following three primary factors: inadequacy of the model architecture, incorrect data representation, and data insufficiency. The deep learning model type has proven itself effective in the industry, enabling the capture of nonlinear relationships that aptly describe the subject area. The data representation developed within the framework of this study yielded high predictive quality, which was confirmed using standard cross-validation techniques. A factor contributing to further accuracy enhancement is the quantity of data, which is expected to accumulate with the proliferation of high-tech diagnostic methods and the prevalence of data management systems.

It should be noted that in this study, we proceeded from the assumption that all genes with mutations are targets. However, in practice, alterations in active signaling pathways often occur even when inhibiting the activity of a key gene. Undoubtedly, the obtained results require validation through laboratory methods.

## Conclusion

The pipeline of methods presented in this paper can serve as the basis for the technology of automated AI-driven drug discovery. The application of modern methods of machine learning, in particular, deep learning, as well as ways to present initial data for learning algorithms, is demonstrated. The performance of the methods, confirmed by cross-validation approaches on known results, was demonstrated using data from open sources. Ways to improve the methodology are the use of more data, including proprietary, as well as a more detailed representation of the original knowledge, in particular—three-dimensional modeling of interacting molecules.

Natural language processing technologies used in this work have shown effectiveness for processing tens of thousands of articles. They can also be similarly used to compile own databases of scientific publications.

## Data Availability

Publicly available datasets were analyzed in this study. These data can be found at: https://gdc.cancer.gov.
